# Phamerator: a bioinformatic tool for comparative bacteriophage genomics

**DOI:** 10.1186/1471-2105-12-395

**Published:** 2011-10-12

**Authors:** Steven G Cresawn, Matt Bogel, Nathan Day, Deborah Jacobs-Sera, Roger W Hendrix, Graham F Hatfull

**Affiliations:** 1 Department of Biology, James Madison University, 820 Madison Dr. MSC 7801, Harrisonburg, VA, 22807 USA; 2 Pittsburgh Bacteriophage Institute and Department of Biological Sciences, University of Pittsburgh, 4249 5th Avenue, Pittsburgh, PA, 15260 USA

## Abstract

**Background:**

Bacteriophage genomes have mosaic architectures and are replete with small open reading frames of unknown function, presenting challenges in their annotation, comparative analysis, and representation.

**Results:**

We describe here a bioinformatic tool, Phamerator, that assorts protein-coding genes into phamilies of related sequences using pairwise comparisons to generate a database of gene relationships. This database is used to generate genome maps of multiple phages that incorporate nucleotide and amino acid sequence relationships, as well as genes containing conserved domains. Phamerator also generates phamily circle representations of gene phamilies, facilitating analysis of the different evolutionary histories of individual genes that migrate through phage populations by horizontal genetic exchange.

**Conclusions:**

Phamerator represents a useful tool for comparative genomic analysis and comparative representations of bacteriophage genomes.

## Background

Bacteriophages represent a numerically vast, highly dynamic, evolutionarily ancient, and genetically highly diverse population [1-3]. Phage genomes are typically small compared to those of their bacterial hosts (ranging from a few to several hundred kilobases) and no longer present significant technical challenges to sequence determination [1]. As genomic sequencing approaches get simpler and cheaper, the availability of individual phage isolates for characterization becomes limiting, a need that can be effectively addressed through integrated research-education programs involving undergraduate and high school student investigators [4,5].

In spite of their relatively small size, phages present significant challenges to accurate genome annotation including gene identification. Two principal issues arise. First, phage genes tend on average to be small (~600 bp), approximately two-thirds the average size of bacterial genes [1,6]. Many of the genes required for virion structure and assembly are relatively large (tape measure genes can be over 6 kbp long), but those in the non-structural genomic segments are small, often shorter than 100 codons. Secondly, phage genomes are replete with genes of unknown function for which no homologues have been described [7-10].

Mosaic architectures are hallmarks of phage genomes, and individual phages can be considered as particular combinations of interchangeable modules, each of which can be present in two or more different genomic contexts [10,11]. In some cases, where the recombination events giving rise to these mosaic structures occurred relatively recently in evolutionary time, mosaicism is apparent through nucleotide sequence comparisons [12-14]. When the events occurred in more remote evolutionary times the evidence of common ancestry is usually no longer apparent at the nucleotide level, but often can be revealed from comparison of the predicted amino acid sequences [15-17]. Such comparisons reveal that individual phage genomes are typically constructed from multiple modules - often corresponding to single genes - each of which has a distinctly different phylogeny [10]. As such, accurate compilations of whole genome phylogenies that reflect the evolutionary history of the entire genome are not possible, and reticulate-based representations are needed to capture this evolutionary complexity [16,18].

The mechanisms giving rise to genome mosaicism are unclear but must accommodate the striking observation that module boundaries correspond closely with gene boundaries, and in some cases, domain boundaries [11,19]. One model invokes homologous recombination events targeted to short conserved boundary sequences between genes, and there is evidence for this in some phage genomes [20,21]. However, there are numerous examples where no conserved boundary sequences are evident, raising the possibility that mosaicism results largely from illegitimate recombination events between randomly chosen partners sharing little or no sequence identity [10,11]. In this second model, correspondence between module and gene boundaries results from the selection for gene function, not from targeting of the recombination events [22].

Comparison of genomes from phages that infect taxonomically diverse hosts typically provides little information into their evolution because of only very limited similarity at either the nucleotide or amino acid sequence level [4,11]. Phages of a common host, however, have the advantage that they are more likely to have been in recent genetic communication with each other and to have exchanged modules in recent evolutionary times [6]. Large sets of phage genomes are now available for several hosts including *Burkholderia *[9], *Bacillus *[23], *Enterobacteriaceae *[24], *Mycobacteria *[6], *Prochlorococcus *and *Synechococcus *[25], *Pseudomonas *[7], and *Staphylococcus *[8], although even these can span enormous genetic diversity [1,6].

A large number of phages that infect *Mycobacterium smegmatis *mc^2^155 have been isolated and a comparative analysis of 80 has been described [4,6,10,14]. Although these are genetically diverse, the diversity is heterogeneous, and phages can be grouped into 'clusters' according to their overall nucleotide sequence relationships [6]. Of the 80 published completely sequenced mycobacteriophage genomes, 75 can be grouped into ten major clusters, seven of which can be further subdivided into subclusters according to the extent of the nucleotide similarities [14]. Five of the genomes have no close relatives and are referred to as 'singletons' [6]. Because the currently sequenced mycobacteriophage genomes under-represent the mycobacteriophage population-at-large, these cluster designations will undergo modifications as new genomes are sequenced [14]. There are, however, numerous examples of genes that are shared between phages of different clusters and whose common ancestry is only apparent from amino acid sequence similarity [4,6,10]. We have proposed previously [4] that mycobacteriophage genes related to each other can be grouped into phamilies (phams) and that mosaic relationships can be analyzed and represented using pham-annotated genomes maps and phamily circles that show the patterns of which phages contain members of particular phams. Although manual or semi-automated approaches are applicable when only small numbers of genomes are analyzed [4], this becomes an impossible task as the number of genomes expands.

We describe here a software program 'Phamerator' that provides bioinformatic tools for both analyzing and representing phage genome mosaicism. The core functionality of Phamerator performs pair wise similarity searches between predicted protein products of a set of phage genomes, and assorts them into phamilies (phams) of related sequences. Genome maps can be displayed that illustrate the relationships between phages at both the nucleotide and amino acid sequence level. Moreover, the evolutionary histories of specific genes can be displayed by phamily circles in which all gene members of particular phams are represented, and for which multiple phams can be compared. We illustrate the utilities of Phamerator using a set of 111 completely sequenced mycobacteriophage genomes, but the program is applicable to any set of phage genomes for which comparative analysis is desired.

## Methods

### Phamerator database architecture

Phamerator is written entirely in the Python computer programming language and makes use of a number of modules, including the Biopython framework for computational biology [26]. Biopython provides a programmatic interface for sequence manipulation, the construction and parsing of files in relevant formats, and access to external command line applications such as those used for sequence alignment. For the latter, Phamerator uses Biopython to interact with local instances of BLASTP and CLUSTALW. Due to the significant computational time required for performing large numbers of sequence alignments, Phamerator employs a distributed processing model that is implemented using Python Remote Objects (Pyro). Phamerator was developed on Ubuntu Linux but should be able to run on any modern UNIX-based operating system.

**Figure 1:**
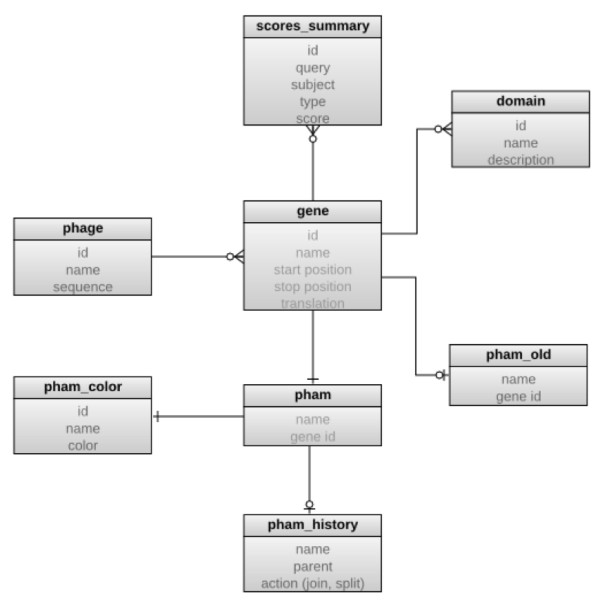
**Database structure**. An entity relationship diagram of the Phamerator database schema. Boxes represent SQL database tables, with table names in bold and column names in gray. The *gene *is the central element of the design, with the domain and pham tables storing data related to individual genes. The *pham_history *and *pham_old *tables record information regarding the automatic joining or splitting of phams as genomes are added or removed from the database.

Phamerator uses the MySQL database software with a simple, custom database schema that incorporates and extends the relevant information found in GenBank records. The *phage *and *gene *tables are populated with data from GenBank files, while the remainder store data relevant to Phamerator analysis or imported data from external databases such as the NCBI conserved domain database (Figure 1). Additional tables are used to store current and historical pham assignments, and records are maintained of the splitting or joining of phams that can occur as new sequences are added to the database. In the event that a new mycobacteriophage protein is added to the database that has similarity to members of more than one existing pham, the phams are merged, their pham names (numbers) retired, and a new pham created with a new number. The new pham contains each of the members of the joined phams in addition to the new protein. Conversely, the addition of new proteins to the database can also invalidate an existing pham because the BLASTP E values used for determination of pham membership are in part dependent on the size of the data set. Thus, after adding a new genome to the database, if a protein in a pham is no longer related to any members of the pham it is removed from the pham, and it is either placed into an existing pham if that pham includes a related protein, or it becomes an orpham (a pham containing only a single member).

### Use of distributed computing resources

Adding genomes to a large Phamerator database is time-consuming, with the length of time required being proportional to the size of the existing database and the number of genes in the genome to be added. However, the length of time required to perform this operation can be reduced by distributing the required calculations to several computers. The reduction in time required scales approximately linearly with the number of available computers. Communication between computers is achieved using the Python Remote Objects (Pyro) library, and can be distributed to computers on the local network or across the Internet.

### Availability and distribution

Phamerator operates using a client/server model. A relational database resides on the server computer and is distributed to client computers when they run the Phamerator client program. This enables all clients to have the performance benefits associated with interacting with data stored on the local computer along with a benefit normally associated with processing data on a server--assurance that the data being used is up-to-date. Because the data files being disseminated are relatively small (approximately 10-100 megabytes), a modest server can handle the load of hundreds of users without performance deterioration.

Phamerator can be downloaded from: http://phamerator.csm.jmu.edu/files/phamerator-current.tar.gz. Further information and installation instructions are provided in Additional Files 1 and 2.

## Results

### Rationale for Phamerator construction and operations

The pervasive mosaicism of bacteriophage genomes requires bioinformatic tools that can organize and display their complex relationships. Two key questions arise in phage comparative analysis: what are the relationships between genes that are evolutionarily mobile within a given set of phage genomes, and how are they related to genes found in other genomes. Both approaches are complicated by the presence of intragenic mosaicism reflecting distinct evolutionary histories of gene segments [4,13,27-29].

Phamerator is a computational tool designed to sort phage genes into phamilies of related sequences using pairwise amino acid sequence comparisons of predicted gene products. Rather than using ortholog identification programs such as HMMER or Pfam [30] we have employed BLASTP and CLUSTALW to perform pairwise comparisons that are then assembled into phamilies of related proteins. Both of these programs are relatively fast computationally, a critical factor when large numbers of computations are required. For example, in the dataset of 111 phage genomes used here, a total of 1.77 × 10^8 ^comparisons must be computed. We note that the use of efficient pairwise BLASTP comparisons to generate families of protein sequences has been described previously [31].

To identify homologues of previously identified proteins Phamerator performs automated searches of GenBank non-redundant protein sequences, as well as searches for conserved domains in the NCBI conserved domain database using the RPS-BLAST tool. This information can be exported in tabular form, or represented in a whole-genomic context. Details of the Phamerator program are described in the Methods section and an overview of database structure is shown in Figure 1.

### Pham-building parameters

The building of phams is strongly influenced by the specific parameters used for amino acid comparisons. In early studies initiated prior to Phamerator development we used a BLASTP cutoff value of 0.001 and a CLUSTALW cutoff of 25% amino acid sequence identity for manual pham building [4]. However, as the number of mycobacteriophage genomes increased and computational processing became essential, it became clear that these largely arbitrarily chosen parameters promoted assembly of many large phams that require time-consuming manual deconvolution [6]. We therefore explored the impact of varying the threshold values for BLASTP and CLUSTALW on pham assembly.

**Figure 2:**
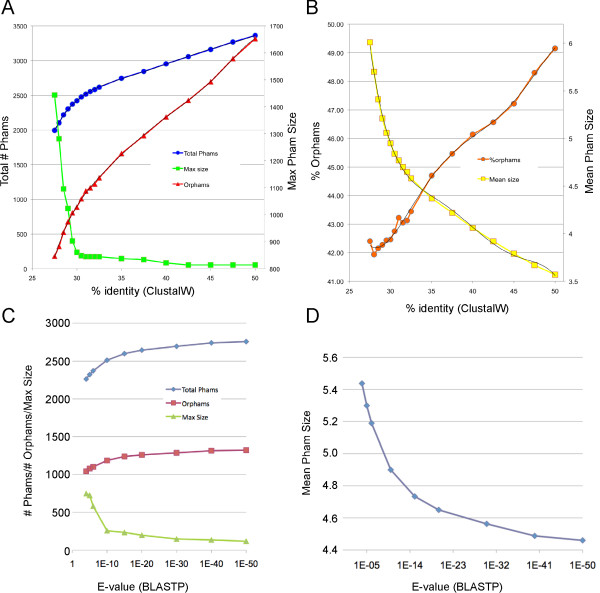
**Effects of CLUSTALW and BLASTP thresholds on pham assembly**. A. Changes in the total number of phams, number of orphams, and maximum pham size as a function of CLUSTALW threshold (percent identity). B. Changes in the percent of total phams that are orphams and mean pham size as a function of CLUSTALW threshold (percent identity). C. Changes in the total number of phams, number of orphams, and maximum pham size as a function of BLASTP threshold (E-value), superimposed over a CLUSTALW cutoff value of 32.5% identity. D. Change in the mean pham size as a function of BLASTP threshold (E-value), superimposed over a CLUSTALW cutoff value of 32.5% identity.

We first evaluated the effect of changing the threshold for CLUSTALW comparisons. We varied the threshold between 50% amino acid sequence identity and 27.5%, and for each level determined the number of phams generated, the size of the largest pham, the number of orphams (single-member phams), the percentage of orphams, and the mean pham size (Figure 2A, B). These data are informative and provide guidance as to the optimal parameters to use for routine database construction. In particular, we note that as the threshold for amino acid sequence similarity is made less stringent (50% to 27.5% identity) there is a reduction in the total number of phams (from 3, 363 to 1, 995) reflecting the process of pham assembly. Interestingly, this relationship is linear between 50% and 32.5%, with a reduction in the total pham number of about 40 for every percentage of identity that is reduced (Figure 2A). As the percent identity falls below 32.5% the relationship becomes non-linear, with a progressively greater reduction in the number of phams as the percent identity threshold falls from 32.5% to 27.5% (Figure 2A). The number of orphams also reduces as stringency is relaxed, while mean pham size increases as stringency is relaxed, and there are notable changes between values above and below 32.5% identity (Figures 2A, B).

There is also a dramatic change in the size of the largest pham as the threshold level varies from 32.5% to 27.5% (Figure 2A). At 32.5% the largest pham contains 172 members, but increases to 2, 505 at 27.5% (Figure 2A). The size of the largest pham is more stable between 32.3% and 50% identity thresholds and varies from 172 to 53. We interpret these data as indicating that between 50% and 32.5% identity, pham assembly proceeds in a manner that simply reflects the variation in the overall relationships between genes. However, at levels below 32.5%, there is an increasing proportion of phams that are more complex, such that not all pairwise matches within the pham are above the threshold level. One example might be where two genes (e.g. gene A and gene B) have been fused into a single open reading frame (gene C), such that although genes A and C, and gene B and C, both surpass the threshold, the unrelated genes A and B do not (a specific example is phage PBI1 genes *6 *and *7*, which are fused in phage Gumball to form gene *6*). For routine database building purposes where we wish to avoid the assembly of large phams that then warrant subsequent deconvolution, we have chosen to use a 32.5% CLUSTALW threshold, but note that comparison of phams generated with these parameters and those with lower stringencies should be useful in analyzing intragenic mosaic relationships.

One advantage of CLUSTALW as an assembly program is that the threshold values are independent of gene length. Nonetheless, we predict there are instances where large genes may not exceed the CLUSTALW threshold but are evidently homologues because of statistically informative BLASTP scores. We therefore examined the impact of including a BLASTP search along with the CLUSTALW comparison (using a 32.5% cut off value) and varying the BLASTP cut off value (pham membership thus required meeting either the CLUSTALW or the BLASTP thresholds) (Figure 2C, D). As threshold values are made less stringent we observe a reduction in the total number of phams and the number of orphams, and a corresponding increase in mean pham size and the size of the largest pham (Figures 2C, D). Between BLASTP thresholds of 10^-50 ^and 10^-20 ^these is only a modest change in the total pham number (from 2, 757 to 2, 644; ~4% reduction) and number of orphams (from 1, 322 to 1, 260; ~4% reduction), but the size of the largest pham changes from 118 to 198 (68% increase). Upon manual inspection of all phams with 100 or more members at the BLASTP thresholds between 10^-50 ^and 10^-20 ^we see 'false' pham assembly occur as illustrated by joining of a phamily of tape measure proteins with a phamily of lysin proteins, through sharing of small but closely related domains. Nonetheless, inclusion of the BLASTP comparison with a 10^-50 ^threshold joins several lysin phams that are separate when using just the CLUSTALW comparison. We conclude that inclusion of BLASTP contributes rather little to the pham assembly process, but that a combination of a CLUSTALW threshold of 32.5% and a BLASTP cut off of 10^-50 ^offers optimal parameters for this dataset, building phams of homologous proteins while minimizing construction of complex phams in which only segments of the proteins are related to each other.

When these parameters are applied to this dataset, the 111 mycobacteriophage genomes contain a total of 12, 298 genes that assemble into 2, 757 phamilies with a mean size of 4.46 genes/pham; 1, 322 phams are orphams (48%) and the largest pham contains 118 members.

### Identification of known homologues and conserved domains

Once a novel genome has been sequenced and annotated, questions about the functions of individual genes encoded within the genome can be addressed. This process is facilitated by analyzing the predicted gene products for the presence of conserved domains. Numerous tools already exist for this purpose, but the NCBI conserved domain database (CDD) aggregates many of them into a single, searchable dataset. These domain databases often use different, complementary techniques such as hidden Markov models or position-specific scoring matrices to define domains and for the matching of novel sequences to existing domain models. To leverage the power of each of these approaches, we have implemented a system in Phamerator whereby phage proteins are used to query CDD, and the results are presented in a searchable database browser in addition to being displayed on genome maps. The latter affords both a global view of the genomes and provides a way to visualize the conservation of specific sequences in the Phamerator dataset with those in other organisms.

An analysis of the distribution of CDD hits among the mycobacteriophage protein sequences reveals that a current search produces a total of 16, 420 matches among the 18, 901 predicted proteins, for an average of 0.87 matches per protein. However, as expected, the distribution of matches is non-random, with only 2, 981 proteins (15.8%) having at least one CDD entry match, with the average number of matches for these proteins being 5.51. While 935 proteins each match a single domain, a single protein, Myrna gp183 (the presumed Lysin A), matches 77 domain models, most of which are aminotransferases. Generally, when numerous matches of a single protein to domains in the CDD are found, it reflects the redundant nature of the CDD dataset, although in some cases it reflects the presence of multiple domains within a single protein.

### Computation of nucleotide sequence similarities

Phamerator uses the BLAST "Align Two Sequences" program (bl2seq) to perform pairwise local alignments of whole genome sequences. An E value of 1e-4 was chosen as the BLAST threshold. The alignments are performed between adjacent genomes on the linear genome maps and the results are overlaid on the maps using shading between the genomes to depict the aligned regions. This shading is color-coded according to the E value with violet representing an E value of zero and red an E value equal to the threshold used.

### Representation of genome maps

**Figure 3:**
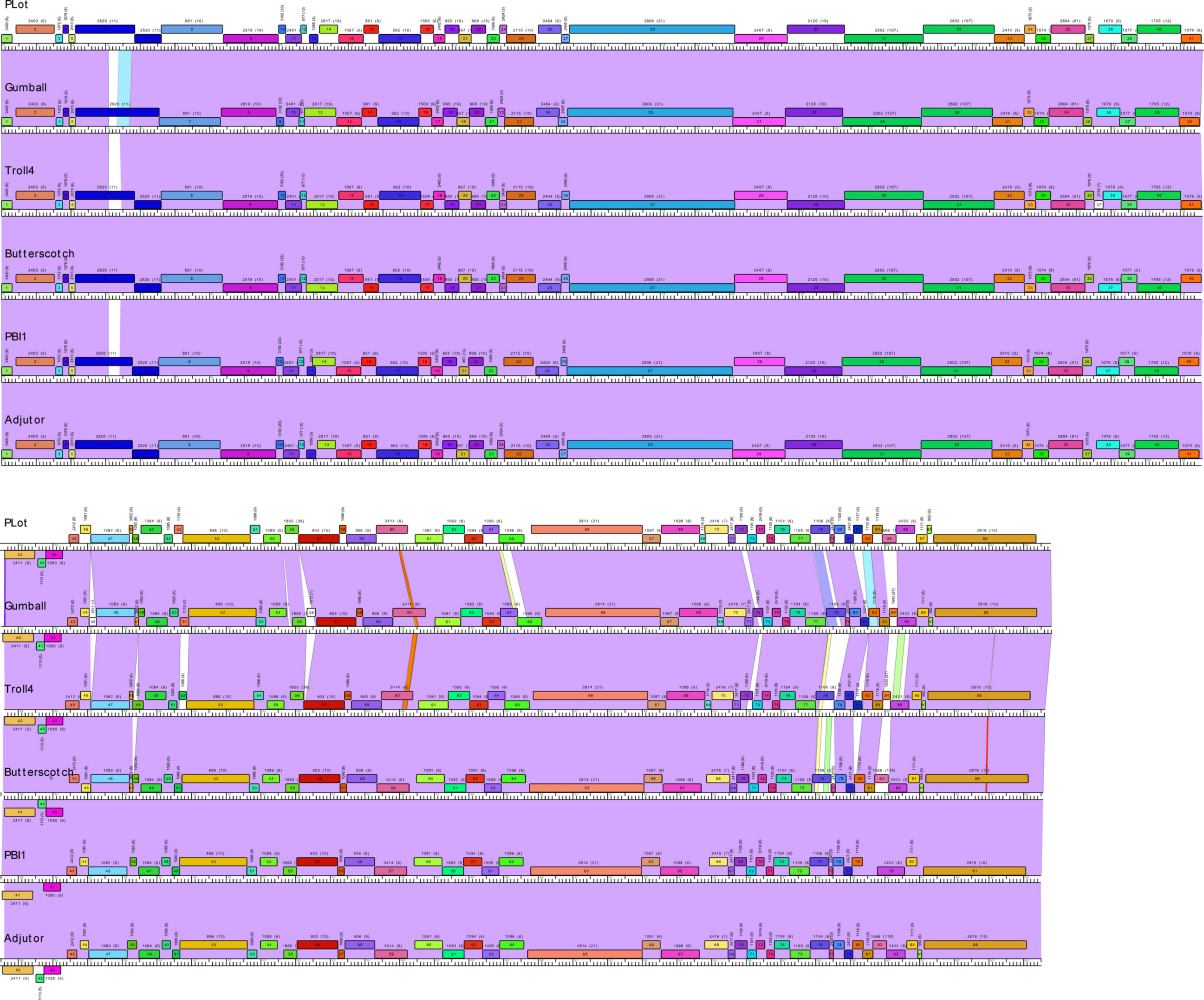
**Phamerator-generated genome maps**. A. Genome maps of six Cluster D phages (Plot, Gumball, Troll4, Butterscotch, PBI1 and Adjutor). The genomes are shown in two tiers. Genes are color-coded according to their pham assignment. Gene numbers are shown within each gene box, and the pham number and number of pham members in parentheses shown above each gene. Pairwise nucleotide sequence similarities are presented as colored shading between genomes; color spectrum reflects the extent of nucleotide sequence similarities with violet being the most similar and red being the least similar. No shading shows that there is no similarity with a BLASTN score of 10^-4 ^or better.

Perhaps the key functionality of Phamerator is the construction of phage genome maps that incorporate the nucleotide similarity and pham assignment information. An example of this is shown in Figure 3. When the six genomes that currently constitute Cluster D are displayed, each is represented by a horizontal bar with coordinate markers with putative genes shown as colored boxes either above or below, corresponding to rightwards or leftwards transcription respectively (Figure 3). The name of each gene is shown within the gene box, and the pham number is shown above with the total number of pham members shown in parentheses. Each pham has a designated color, with the exception of orphams that are shown as white boxes.

**Figure 4:**
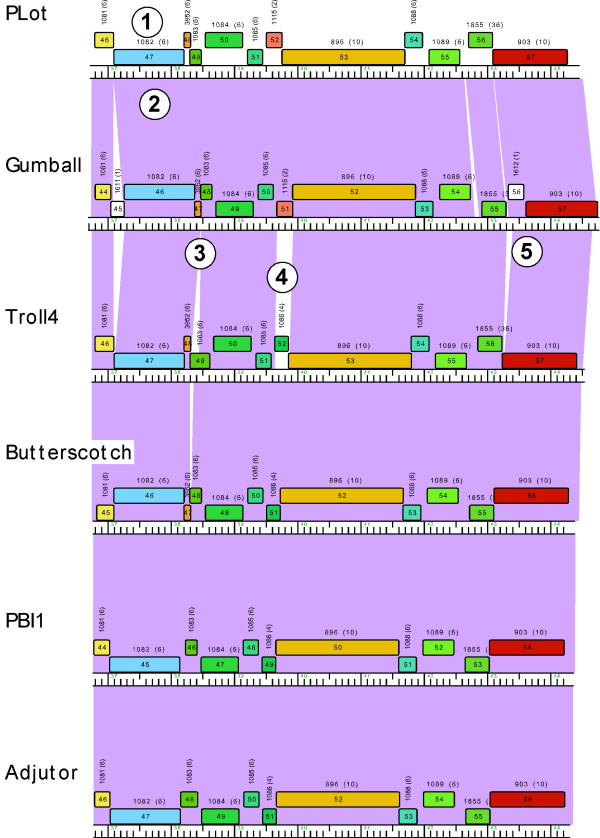
**Expanded view of Cluster D genome maps**. Five specific features are indicated. Feature #1 shows the designation of the pham assignment (Pham1082) for Plot gene *47*, and that Pham1082 contains six members (shown in parentheses). The six genomes shown all contain a member of Pham1082, and thus there are no other members of Pham1082 outside of Cluster D. Feature #2 shows the violet shading between Plot and Gumball genomes, reflecting a high degree of nucleotide sequence similarity. Feature #3 illustrates a departure in the synteny of phages Gumball and Troll4, with an apparent insertion within Troll4 gene *49*, relative to Gumball gene *48*, both of which are in Pham1083. Feature #4 indicates a replacement of Gumball gene *51 *for the Troll4 gene *52*, reflected in the lack of nucleotide similarity and the designation of the genes in two different phams (Pham1115 and Pham1086 respectively). Note that PLot shares a member of Pham1115 and Butterscotch, PBI1 and Adjutor share members of Pham1086. Feature #5 shows a small insertion in Gumball relative to Troll4 (as well as Butterscotch, PBI1 and Adjutor) that leads to an alternative annotation of this genome segment, with inclusion of a putative new orpham (Gumball gene *56*) and shorter version of Gumball gene *57*.

**Figure 5:**
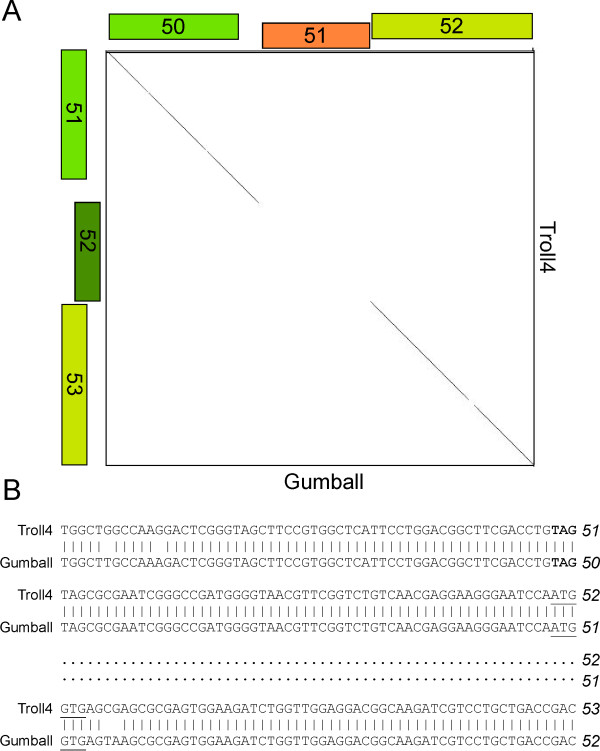
**Lack of nucleotide similarity between Gumball gene *51 *and Troll4 gene *21***. A. Dotplot comparison of Gumball genes *50*-*52 *and Troll4 genes *51*-*53 *(see feature #4 in Figure 4). B. Alignment of DNA segments of Troll4 and Gumball shows that the boundary of sequence identity and non-identity occurs precisely at the beginnings of Troll4 gene *52 *and Gumball gene *51 *(the ATG start codons are underlined) and the beginnings of Troll4 gene *53 *and Gumball gene *52 *(GTG start codons are underlined).

Because the genomes shown in Figure 3 are all members of the same cluster they share substantial nucleotide sequence similarity, which is reflected by the extensive violet shading between adjacent genomes in the stack of maps. Genomes can be easily re-positioned both vertically and horizontally within the display such that different pairwise relationships can be captured. For genomes within a designated cluster - such as those in Figure 3 - interruptions in the nucleotide sequence similarity are readily apparent, seen as either a reduced level of similarity (by shading with colors towards the red end of the spectrum) or by no shading (reflecting absence of DNA similarity below a BLASTN cut off value of 10^-4 ^using the Align Two Sequences algorithm. For example, in Figures 4 and 5 comparison of Gumball and Troll4 reveals a mosaic substitution of Troll4 gene *52 *with Gumball gene *51*, with the flanking sequences being very closely related. PLot shares the same organization as Gumball, whereas Butterscotch, PBI1 and Adjutor all share the Troll4 organization. The different segments of DNA also encode proteins of different sequences, because the predicted genes belong to different phams [Pham1115 (Gumball) and Pham1086 (Troll4)]. Dotplot analysis clearly shows that Gumball gene *51 *and Troll4 gene *52 *segments are unrelated at the nucleotide level (Figure 5A) and sequence alignment reveals that the discontinuities occur at the start codons of these genes, and those of the downstream genes (Figure 5B). The map function of Phamerator provides a tool for readily identifying and analyzing these module boundaries.

**Figure 6:**
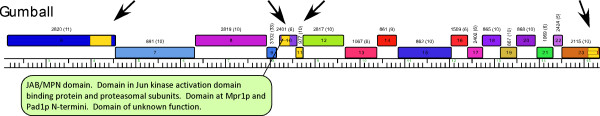
**Representations of conserved domains**. A segment of the Gumball genome is displayed while using the Show Conserved Domains functions in Phamerator. Within the gene *6 *- *23 *region there are four genes (arrowed) for which conserved domains are displayed, shown as yellow boxes. In genes *6 *and *11*, only a single domain is identified, whereas in genes *10 *and *23*, two and three domains are displayed. These correspond to the same parts of the proteins and therefore reflect redundancy in the CDD database. Holding the mouse over a domain activates a pop-up displaying the domain information, illustrated for a domain in gene *10*.

Phamerator-generated maps optionally can also display conserved domains identified with the automated CDD function (Figure 6). Domain hits are shown as yellow boxes or lines (if there are multiple separate domain hits) within each gene box. Hovering the mouse over any domain pops up a description of that domain hit (Figure 6).

### Phamily circle representations of gene phylogenies

**Figure 7:**
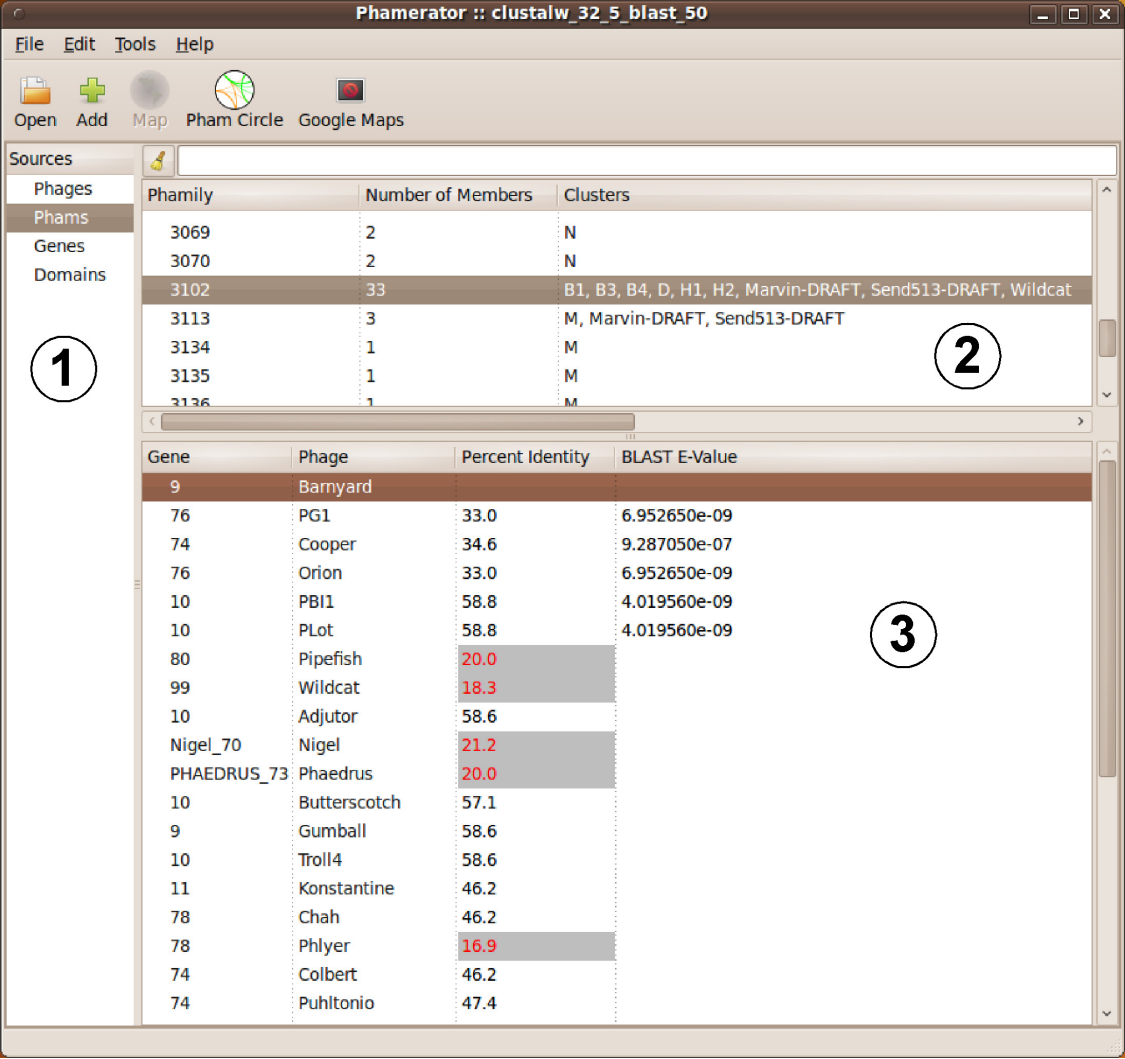
**The Phamily display function of Phamerator**. A screen-shot of the main Phamerator display shows four sources listed in the left-hand panel (feature #1). When the Phams function is selected, a list of all of the phamilies, the numbers of members, and the clusters to which the parent genomes belong are displayed in the top right panel (feature #2). When a particular pham is selected (Pham3102 is shown), the gene members, the parent phages, and the percent identities and BLASTP E-values are shown in the bottom right panel. When a specific gene is selected (Barnyard gene *9 *is shown; feature #3), the percent identity and BLASTP E-values displayed are in reference to the selected gene. The values in red and gray-highlighted are below the threshold values for pham assembly.

**Figure 8:**
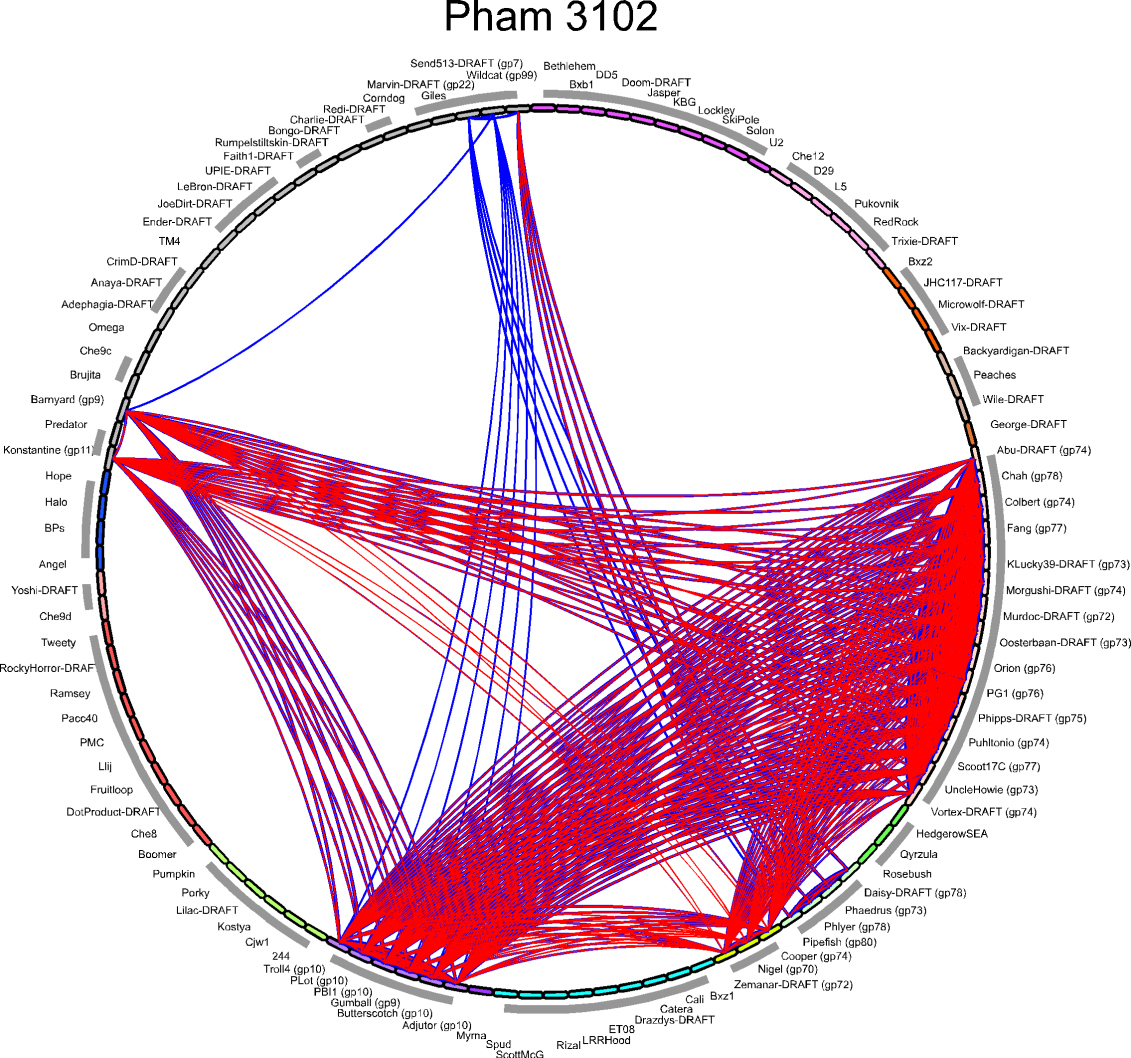
**The Phamily circle representation function**. When the Pham Circle function is chosen (shown in the very top panel in Figure 7), a phamily circle is drawn in which all of the component phages in the dataset are represented around the circumference of a circle, ordered according to their cluster and subcluster designations. An arc is drawn between members of that pham that are related to each other above the threshold values; blue and red arcs show CLUSTALW and BLASTP matches respectively. Some of the relationships only report BLASTP scores, such as the blue arcs between PLot and Send513, and others only CLUSTAL score such as the red arcs between Konstantine and Nigel. Most show red and blue arcs superimposed. Arc widths reflect the strengths of the relationships.

Phamily circles provide a graphic way to illustrate the relationship between proteins in a phamily, and to display which genomes within the dataset contain members of that pham (Figures 7 and 8). The strength of the pairwise relationships for a particular phamily can be simply accessed from the 'Phams' function in the left hand panel (Figure 7). The 'Phams' window displays two separate panels with the upper one showing a numerical list of phams, the number of phamily members, and the clusters and subclusters that are represented. Selecting a pham directs a display in the lower window of each of the phamily members by gene name and phage. When a gene name is selected, Phamerator reports the CLUSTALW and BLASTP score of each of the other members relative to the selected gene (Figure 7).

Phamily circle diagrams can be generated for individual phams and include the name for each phage in the database positioned around the circumference of a circle, ordered and colored according to cluster and subcluster designation (Figure 8). If a given phage has a gene that is a member of the phamily represented in the diagram, the protein name is included with the phage name. Arcs are drawn between pairs of genomes that contain a gene member of that phamily; relationships derived from CLUSTALW analyses are represented in blue, and BLASTP in red. In the Pham3102 example shown in Figure 8, the phamily of small proteins is present in 33 of the genomes and distributed among several cluster and subclusters. Some of the relationships are shown as blue-only arcs, indicating that the relationships exceed the threshold of 32.5% amino acid sequence identity of the CLUSTALW comparisons but does not meet the E value of 10^-50 ^for the BLASTP comparisons (Figure 8). The gene sequences can be readily exported for each pham and used to construct neighbor-joining trees for comparison with the phamily circles.

### An abundance of Orphams

**Figure 9:**
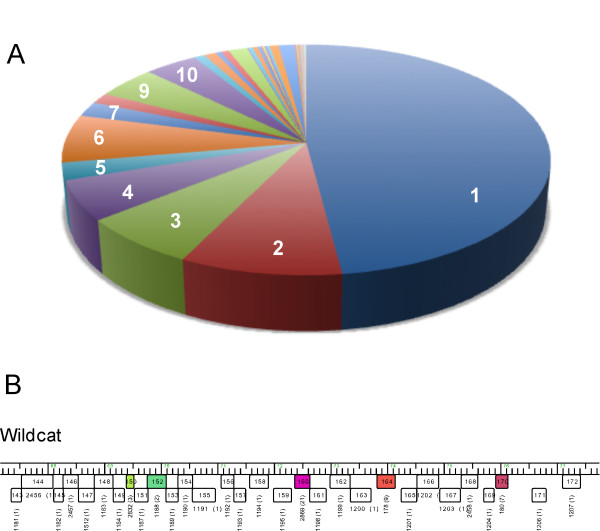
**Distributions of pham sizes**. A. The proportions of phams containing a single member (i.e. orphams), two members, or more - as indicated by the white numbers - are represented as a pie chart. B. A segment of the genome of the singleton phage Wildcat shows the abundance of small genes of which many - shown as white boxes - are orphams.

The great genetic diversity of the mycobacteriophage population is reflected in the large number of orphams (1, 322; 48%), the relatively low mean and median pham sizes (4.46 and 2 genes respectively), and the observation that 91% of the phams contain ten or fewer members (Figure 9A). The question arises as to whether the orpham designation is useful, because one gene member might be deemed insufficient to form a phamily. We think this is a useful designation because this is an especially informative group when attempting to identify those genes that have been most recently acquired in evolutionary time [6], but also note that orphams are especially abundant in singleton genomes for which no close relatives have yet been isolated (Figure 9B). The abundance therefore reflects the current poor sampling of the mycobacteriophage population, and as relatives of the current singleton genomes are discovered, many of the orphams are anticipated to be assembled into larger phams [6]. We anticipate a substantial reduction in the proportion of orphams as we approach saturation of the phage population, but it is not yet simple to predict when that will occur.

## Discussion

We have explored the use of Phamerator with several other sets of phage genomes including SPO1-like phages of *Bacillus subtilis *[23] and a group of *Streptomyces *phages, but we have recently successfully generated a database from 319 genomes, substantially larger than the 111-genomes described here. We recognize that as the number of complete phage genome sequences increases that the computational time required increases as the square of the number of genes, and this could impose considerable limitations. For example, increasing the number of genomes to 1, 000 - not an unreasonable expectation given the advances in DNA sequencing technology - increases the number of pairwise computations to ~10^11^, a 1000-fold increase in time over the current dataset. However, with recent advances in cloud computing and the availability of massively parallel and multi-core computing systems we anticipate that these demands can be readily met. For example, cloud-computing systems can provide more than a 1000-fold increase in the number of processors at minimal cost. Phamerator will remain a useful tool for comparative phage genome analysis for the next few years. We also note that recent developments in alternative profile-based similarity searches such as HMMERHEAD and HHMER3 http://hmmer.org/ that greatly increase their efficiencies should provide additional Phamerator components [32,33].

## Conclusions

Phamerator provides a simple but useful computational tool for dissecting the genetic relationships among bacteriophage genomes, and displaying them in informative representations. Phamerator is especially useful for analysis of particular sets of phages such as the mycobacteriophages described here, but can be readily expanded to include a broader phage set, in which it is desired to map the horizontal exchange of genes between phage populations (for example, between *Streptomyces *phages, *Propionibacterium acnes *phages, *Rhodococcus *phages, and the mycobacteriophages). Because of its computational intensity, it is less well suited to mapping global genome-scale relationships among large phage genome sets, but other programs have been described for this purpose [34,35]. The use of a commonplace Biopython framework and MySQL database software should facilitate interaction of the Phamerator database components with other web-based utilities to make this a broadly accessible utility.

## Competing interests

The authors declare that they have no competing interests.

## Authors' contributions

SGC. wrote the Phamerator code with assistance from MB and ND DJS, RWH, and GFH contributed to the Phamerator design and its applications. SGC, RWH, DJS and GFH wrote the manuscript. All authors read and approved the final manuscript.

## Supplementary Material

Additional file 1 **Phamerator program**. Phamerator program. Click here for file

Additional file 2 **Phamerator Installation Instructions**. This file contains installation instructions for Phamerator. Click here for file
