# Supplementary figures and images for: Phamerator: a bioinformatic tool for comparative bacteriophage genomics
Source: BMC Bioinformatics. 2011 Oct 12;12:395. doi: 10.1186/1471-2105-12-395 (PMC3233612; doi:10.1186/1471-2105-12-395)

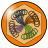

Supplement: Additional file 1 — Phamerator program. Phamerator program. [file 1471-2105-12-395-S1.GZ › phamerator-1.1/phamerator/pixmaps/phamerator2.png]

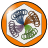

Supplement: Additional file 1 — Phamerator program. Phamerator program. [file 1471-2105-12-395-S1.GZ › phamerator-1.1/phamerator/pixmaps/phamerator.png]

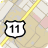

Supplement: Additional file 1 — Phamerator program. Phamerator program. [file 1471-2105-12-395-S1.GZ › phamerator-1.1/phamerator/GMaps_round.png]
