# Supplementary material for: Phamerator: a bioinformatic tool for comparative bacteriophage genomics
Source: BMC Bioinformatics. 2011 Oct 12;12:395. doi: 10.1186/1471-2105-12-395 (PMC3233612; doi:10.1186/1471-2105-12-395)
Supplement: Additional file 2 — Phamerator Installation Instructions. This file contains installation instructions for Phamerator. [file 1471-2105-12-395-S2.DOC]

**Installing and Running Phamerator**

***Getting Started:***

Phamerator is developed on Ubuntu Linux 10.10, but should run on any modern distribution of Linux. It does not run directly on Microsoft Windows or Mac OS X. However, you can run Linux on Windows or Mac OS X in a virtual machine using software such as VirtualBox (available for free at [http://virtualbox.org](http://virtualbox.org/)). Both VirtualBox and VMWare Fusion (for the Mac) work well in our experience.

***Preparing to Run Phamerator:***

Each of the remaining steps should be performed within the Linux environment.

Several freely available software packages for Linux are required in order to run **Phamerator**, and you must first download these if you don’t already have them.

These programs are listed below according to their package names on Ubuntu. Package names on other distributions of Linux are similar.

- pyro
- clustalw
- python-biopython
- mysql-server
- python-mysqldb
- python-pygoocanvas
- curl

These packages can be installed with the following command on Ubuntu (if copy/pasting, please ensure that when pasted this command is on a single line):

sudo apt-get install pyro clustalw python-biopython mysql-server python-mysqldb python-pygoocanvas curl

If you have not installed the mysql-server database software previously, you will be prompted to set up a password for the root account on the database. (You will need this password when you run Phamerator for the first time, or any time you add a new Phamerator database.)

***Setting up the Phamerator database***

Download Phamerator using your web browser from the following address:

<http://phamerator.csm.jmu.edu/files/phamerator-1.1.tar.gz>

Extract the phamerator-1.1.tar.gz file:

tar xzvf phamerator-1.1.tar.gz

***Running Phamerator***

To run Phamerator, change to the directory that was created when you extracted the phamerator-1.1.tar.gz file.

cd phamerator-1.1/phamerator

Then type the following command.

./Phamerator

If you have installed the mysql-server database software previously, you will be required to enter your root database password when you run Phamerator for the first time.

The default database for this version of Phamerator is called *SEA*. Please allow a few minutes for Phamerator to download and install the SEA database when you run it for the first time.
